# Muscle Activation during Gait in Children with Duchenne Muscular Dystrophy

**DOI:** 10.1371/journal.pone.0161938

**Published:** 2016-09-13

**Authors:** Juliette Ropars, Mathieu Lempereur, Carole Vuillerot, Vincent Tiffreau, Sylviane Peudenier, Jean-Marie Cuisset, Yann Pereon, Fabien Leboeuf, Ludovic Delporte, Yannick Delpierre, Raphaël Gross, Sylvain Brochard

**Affiliations:** 1 CHRU de Brest, service de pédiatrie, Brest, France; 2 Laboratoire de Traitement de l'Information Médicale INSERM U1101, Brest, France; 3 CHRU de Brest, Service de Médecine Physique et Réadaptation, Brest, France; 4 L'Escale, service central de rééducation pédiatrique, Lyon, France; 5 CNRS, UMR 5558, Pierre-Bénite, France; 6 CHRU de Lille, Service de médecine physique et de réadaptation, Lille, France; 7 CHRU de Lille, service de neurologie pédiatrique, Lille, France; 8 Centre de Référence Maladies Neuromusculaires Nantes-Angers, CHU de Nantes, Nantes, France; 9 Atlantic Gene Therapy Institute, Nantes, France; 10 CHU de Nantes, Pôle de Médecine Physique et Réadaptation, Nantes, France; 11 Plateforme « Mouvement et Handicap », Hospices Civils de Lyon, Bron, France; 12 Service de rééducation neurologique pédiatrique, centre de l'Arche, Le Mans, France; Rutgers University Newark, UNITED STATES

## Abstract

The aim of this prospective study was to investigate changes in muscle activity during gait in children with Duchenne muscular Dystrophy (DMD). Dynamic surface electromyography recordings (EMGs) of 16 children with DMD and pathological gait were compared with those of 15 control children. The activity of the rectus femoris (RF), vastus lateralis (VL), medial hamstrings (HS), tibialis anterior (TA) and gastrocnemius soleus (GAS) muscles was recorded and analysed quantitatively and qualitatively. The overall muscle activity in the children with DMD was significantly different from that of the control group. Percentage activation amplitudes of RF, HS and TA were greater throughout the gait cycle in the children with DMD and the timing of GAS activity differed from the control children. Significantly greater muscle coactivation was found in the children with DMD. There were no significant differences between sides. Since the motor command is normal in DMD, the hyper-activity and co-contractions likely compensate for gait instability and muscle weakness, however may have negative consequences on the muscles and may increase the energy cost of gait. Simple rehabilitative strategies such as targeted physical therapies may improve stability and thus the pattern of muscle activity.

## Introduction

Duchenne Muscular Dystrophy (DMD) is the most common neuromuscular disease in children, with an incidence of 1 in 3500 male births [1]. It causes a lack of dystrophin in the muscles, leading to a progressive degeneration of muscle fibres and generalised muscle weakness [2]. Gait begins to deteriorate between the ages of 3 and 6 years [3] and is lost at around 10–12 years of age [4]. Gait loss is a milestone in the progression of the disease.

Case-control studies using optoelectronic systems to analyse gait have shown kinematic anomalies at the pelvis (lumbar hyperlordosis, excessive anterior pelvic tilt, double bump pattern), the knee (loss of knee flexion in stance and excessive extension at the end of stance) and the ankle (increased plantarflexion in swing, toe-walking) [3,5–14]. These pathological patterns are the result of a slow, poorly-understood process caused by a combination of muscle weakness, muscle shortening and osteo-articular changes, along with postural adaptations.

The underlying patterns of muscle activity have, however, been little studied. There is thus a lack of understanding of the specific role of each lower limb muscle in the abnormal gait patterns. In 1981, Sutherland et al. [5] found abnormal activation of the hamstrings (HS), tibialis anterior (TA) and gastrocnemius-soleus (GAS) muscles. More recently, Patte et al. found excessive and/or prolonged activity of the gluteus maximus, rectus femoris (RF), HS and TA while the activity of other muscles, such as GAS, was close to normal [11]. However, the description of electromyographical (EMG) activity in the literature is generally limited because it was not the primary aim of these studies, and most used qualitative methods. In DMD, the efferent motor command is considered to be intact, thus changes in muscle activity during gait are the direct result of the disease on the muscles. Analysis of muscle activation during the gait cycle as well as intra-segment agonist/antagonist coactivation would be useful to identify “dynamic” muscle adaptations to compensate for weakness and stabilise gait. Knowledge of these adaptations would help to guide gait rehabilitation as well as all types of medical and surgical interventions (splinting, surgery, intra-muscular treatments etc.). It would also help to determine whether alternative rehabilitative strategies decrease compensatory muscle activity.

The primary aim of this study was to compare muscle activation and coactivation in the lower limbs during the gait cycle in children with DMD with typically developing children of the same age. We hypothesised that abnormal patterns of muscle activity would be found in the different segments of the lower limbs in the children with DMD. The secondary aim was to explore the relationship between muscle activation, lower limb kinematics and functional status.

## Research Design and Methods

### Ethics statement

This observational case/control study was approved by the ethics committee “Comité de Protection des Personnes Ouest VI”. The children and parents were given an information letter and signed a consent form for participation in the study, according to French legislation.

### Participants

Children with DMD were recruited in 5 centers specialised in DMD in France who all had optoelectronic gait recording systems (Centre de l’Arche du Mans, CHRU de Brest, CHRU de Lille, CHRU de Nantes, CHRU de Lyon). The criteria for inclusion in the study were: (1) Duchenne Muscular Dystrophy confirmed by dystrophin marking in immunohistochemistry, from muscle biopsy and/or mutation of the dystrophin gene found in molecular biology, (2) boys <18 years of age, (3) able to walk 10 metres without using aids, (4) pathological gait pattern (marked hyperlordosis, toe-walking, shoulder tilt found during a routine medical consultation. In order to confirm this last criterion, the children underwent gait analysis and were included if they had a Gait Profile Score (GPS) which deviated by more than 1.6° (on the left or right) from the mean of the group of healthy subjects. This deviation threshold, calculated by Baker et al. [15] as the Minimum Clinically Important Difference for the GPS, provided a criterion from which ‘pathological gait’ could be objectively determined. Children who had undergone orthopaedic surgery of the lower limbs within the previous 6 months were excluded, as were children with cognitive deficits or behavioural difficulties which would have limited comprehension and participation in the study. Twenty children underwent gait analysis and 16 were included in the final analysis based on their GPS. Fifteen control boys aged from 6 to 13 years with no gait anomalies were also included. The group of children with DMD and the control children were all male and were similar in terms of age and Body Mass Index (Table 1), factors which can influence gait parameters [16].

**Table 1:** Subject characteristics.

	DMD	Control	p value *
Number of children	16	15	
Age (years) (mean and SD)	8.67 (2.04)	9.39 (2.21)	0.42
BMI (kg/m2) (mean ans SD)	16.65 (3.37)	16.07 (1.38)	0.92
Corticotherapy (yes/no)	12 / 4	0	
Vignos Lower Extremity scale (max = 10) (mean and SD)	3.37 (1.36)		

BMI, body mass index.

* p values were calculated using the Mann Whitney test.

### Data collection

The following data were recorded during the clinical examination: medication (particularly corticosteroids), scoliosis, anthropometric parameters (height and weight), Vignos functional rating scale, which classifies children with dystrophinopathies into 10 categories according to their gait capacity [17], hip, knee and ankle flexor and extensor muscle strength using the Medical Research Council scale (MRC scale) from 0 to 5 [18], and passive range of dorsiflexion with the knee flexed to evaluate shortening of the triceps surae (Table 2). All the children had full passive range of motion of the hip or knee (i.e. no contractures).

**Table 2:** Values of selected variables.

	DMD	Control	p value ^a^
GDI	75.0 (9.84)	100.24 (6.87)	***
GPS	9.32 (2.24)	4.58 (0.74)	***
Gait speed (m/sec)	0.78 (0.18)	1.21 (0.13)	***
Cadence (step/min)	116.26 (16.8)	129.68 (8.41)	**
Stride length (m)	0.81 (0.14)	1.13 (0.12)	***
Passive ankle dorsiflexion (degree)	10.94 (10.68)		
MRC Scale Hip Flexion (N = 16)	3.27 (0.81)		
MRC Scale Hip Extension (N = 16)	2.88 (0.96)		
MRC Scale Knee Flexion (N = 16)	3.67 (0.86)		
MRC Scale Knee Extension (N = 16)	4.00 (0.79)		
MRC Scale Ankle dorsi flexors (N = 14)	4.23 (0.98)		
MRC Scale Ankle plantar flexors (N = 14)	4.68 (0.64)		

GDI, gait deviation index; GPS, gait profile score; MRC Scale, medical research council scale.

^a^ p values were calculated using the Mann Whitney test (* p<0.05; ** p<0.01, *** p<0.001).

The 5 movement laboratories used the same standardized acquisition protocol to collect spatio-temporal gait parameters (speed, stride length and cadence) and 3D kinematics of the lower limbs using an optoelectronic system (Vicon MX 13, Oxford metrics, Oxford, UK for 4 laboratories, Motion Analysis, Santa Rosa, CA, USA for 1 laboratory). Sixteen reflective markers were positioned on anatomical reference points according to the protocol by Davis et al. [19]. Muscle activity was recorded using a 16-channel EMG system at a sampling frequency of 1080 Hz (CHRU Brest) or 1000 Hz for the 4 others centres. Pre-amplified surface electrodes were positioned on the rectus femoris (RF), vastus lateralis (VL), medial hamstrings (HS), tibialis anterior (TA) and gastrocnemius-soleus (GAS) according to the SENIAM recommendations [20]. The children walked barefoot, without assistance along a 10 metre long walkway. Five trials were recorded with no instructions regarding gait except to walk at their own comfortable pace.

### Data processing

In order to standardize the analysis, the same researcher carried out all the data processing and analysis for all the children (DMD and control) following receipt of the raw data from the 5 laboratories. As well as spatio-temporal and kinematic parameters, two 3D kinematic indexes were calculated: the Gait Deviation Index (GDI) [21] and Gait Profile Score (GPS) [22] (Table 2). These validated indexes are classically used to evaluate the quality of gait in children. The GDI provides a global index of gait quality and the GPS yields a score for each joint and plane of motion. The GDI is calculated by principal component analysis using all the kinematic data from the lower limbs and is expressed as a score out of 100. Each decrease of 10 points corresponds to a difference of one standard deviation from the control population. The GPS is obtained from the mean of the differences calculated by the Root Mean Square Error (RMSE) of each joint in each motion plane for a given child compared with the control group. It is expressed in degrees relative to the “normal” GPS.

The raw EMG signal of each muscle was processed as follows: it was centred by subtracting the mean of the signal, then rectified by taking the absolute value and filtered (4^th^ order zero lag Butterworth filter, cut-off frequency: 8.9Hz) [23] to obtain a “linear” envelope of the EMG signal. In order to compare the profiles of muscle activity, the linear envelops were normalised by the amplitude of the maximum value of the muscle activity across all the gait cycles of the child (from 0 to 1 on the y-axis) and expressed as a percentage of the gait cycle (temporal normalisation) (0%: beginning of the cycle with initial contact, to 100%: end of cycle on the x-axis). For each muscle of each subject, the EMG profile was defined as the mean of the linear envelope curves of the recorded cycles. Mean linear envelopes and standard deviations were then calculated for both groups (DMD and control).

The KeR-EGI index, recently developed and validated by Bervet et al. [24], was calculated for each child (DMD and control). The calculation follows the same methodology as the GDI. It is based on the Euclidian distance between the EMG pattern of a child with DMD and that of a group of healthy subjects (principal component analysis), out of 100. A score equal to or above 100 indicates normal gait and a decrease of 10 points indicates a deviation of 1 standard deviation from the norm. The KeR-EGI is an index of global muscle activity based on the EMG of all the muscles recorded and does not provide information for individual muscles. In order to quantify the deviation from the norm of each muscle group independently and for each subject, the distance from the norm based on the RMSE was calculated, similarly to the calculation for the GPS. A score without units was obtained for each muscle, which we termed EMG-PS (EMG-Profile Score). It was calculated for the whole of the gait cycle (EMG-PS) as well as for stance phase (EMG-PS StP) and swing phase (EMG-PS SwP). Since the stance and swing phases were different for each child, they were normalized. We termed the mean of these scores for each muscle Global EMG-PS.

**Fig 1:**
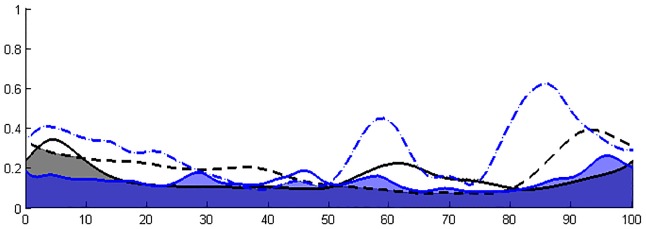
Illustration of co-activation index (CI) calculation. The percentage of the gait cycle is presented on the X-axis (0: heel strike, 100: the following ipsilateral heel strike) and the normalized amplitude of EMG activity on the Y-axis (0–1). Lines represent the mean linear envelope of muscle 1 activation. Dashed lines represent the mean linear envelope of muscle 2 activation. Black color represent muscle activation in the control group. Blue color represent muscle activation in one child with DMD. The calculation index involved overlapping the linear envelopes of muscle 1 and muscle 2, calculating the area of overlap and dividing by the number of data points. Areas of coactivation are represented in grey (control group) and in blue (DMD patient).

Coactivation indices (CI) relating to the timing of simultaneous activation of 2 intra- or inter- segment muscles were calculated following the method described by Unnithan et al. [25] for 3 agonist-antagonist muscle pairs (RF/HS, VL/HS, GAS/TA) for both lower limbs. This coactivation index is obtained by calculating the surface of overlap of the envelopes of the muscle pairs, divided by the number of points in the curve (Fig 1).

To summarize, for each child the Ker-EGI, the Global EMG-PS (global index of muscle activity), the EMG-PS (index of muscle activity for each muscle for the whole of gait cycle, stance phase and swing phase) and coactivation indexes were calculated for agonist-antagonist pairs for both lower limbs. The analyses were carried out on the means of the variables for both groups (control and DMD).

### Statistical analysis

BiostaTGV (version 2014) software was used for all the statistical analyses. A Mann Whitney test was used to compare the variables of the control children and the children with DMD. The means of the variables of each lower limb of the children with DMD were compared with the corresponding limbs of the control children.

A Wilcoxon signed rank test for paired data was used to compare variables from the left and right limbs for each child. Since no significant differences were found between the muscle activations of each limb in either group, the results were pooled across sides for clarity and are presented by muscle group. A qualitative comparison of the mean activation curves was carried out in both groups to compare activation times and the profiles of the curves.

In the DMD group, Spearman’s rank correlation was used to test the association between muscle activation (Ker-EGI, global EMG-PS), kinematics (GDI, GPS, walking speed) and functional status (Vignos functional score). The threshold for significance was set to *p* < 0.05.

## Results

### Muscle activation profile

The KeR-EGI of the children with DMD was more than 2 standard deviations lower than that of the control children (76.88 ±16.9 vs 100.10 ±7.90, p<0.001) and the mean EMG-PS of the children with DMD was significantly higher than that of the control children (0.17 ±0.04 vs 0.13 ±0.03, p<0.001) (Table 3).

**Table 3:** Global index of muscle activity (Ker-EGI and global EMG-PS) and index of muscle activity for each muscle for the whole gait cycle, stance phase and swing phase (EMG-PS).

	Gait cycle			Stance phase			Swing phase		
	DMD	Control	p	DMD	Control	p	DMD	Control	p
KeR-EGI	76.88 (16.9)	100.10 (7.90)	***						
EMG-PS global	0.17 (0.04)	0.13 (0.03)	***	0.18 (0.04)	0.13 (0.01)	***	0.15 (0.04)	0.10 (0.02)	***
EMG-PS Rectus Femoris	0.17 (0.06)	0.13 (0.02)	*	0.16 (0.06)	0.11 (0.03)	*	0.18 (0.06)	0.12 (0.03)	*
EMG-PS Vastus Lateralis	0.14 (0.03)	0.13 (0.03)	p = 0.13	0.15 (0.05)	0.12 (0.02)	p = 0.09	0.11 (0.03)	0.10 (0.02)	p = 0.28
EMG-PS Hamstring	0.18 (0.04)	0.15 (0.03)	**	0.18 (0.06)	0.15 (0.03)	*	0.16 (0.05)	0.11 (0.02)	**
EMG-PS Tibialis anterior	0.20 (0.03)	0.14 (0.04)	***	0.18 (0.03)	0.13 (0.02)	***	0.22 (0.05)	0.13 (0.03)	***
EMG-PS Gastrocnemius Soleus	0.17 (0.07)	0.12 (0.03)	**	0.20 (0.06)	0.14 (0.03)	***	0.08 (0.01)	0.04 (0.01)	*

Ker-EGI, Ker-electromyography gait index; EMG-PS, electromyography profile score.

Since no significant differences were found between the muscle activations of each limb in either group, the results were pooled across sides for clarity and are presented for each muscle group (means and standard deviations).

^a^ p values were calculated using the Mann Whitney test (* p<0.05; ** p<0.01, *** p<0.001).

Mean EMG-PS and EMG-PS by phase were significantly higher in the children with DMD for the RF, HS, GAS and TA muscles. There was no difference between groups for the VL. Activity of the TA (EMG-PS +0.05, p<0.001) and GAS (EMG-PS +0.06, p<0.001) was the most pathological in stance, and activity of the TA was the most pathological in swing (EMG-PS +0.09, p<0.001).

**Fig 2:**
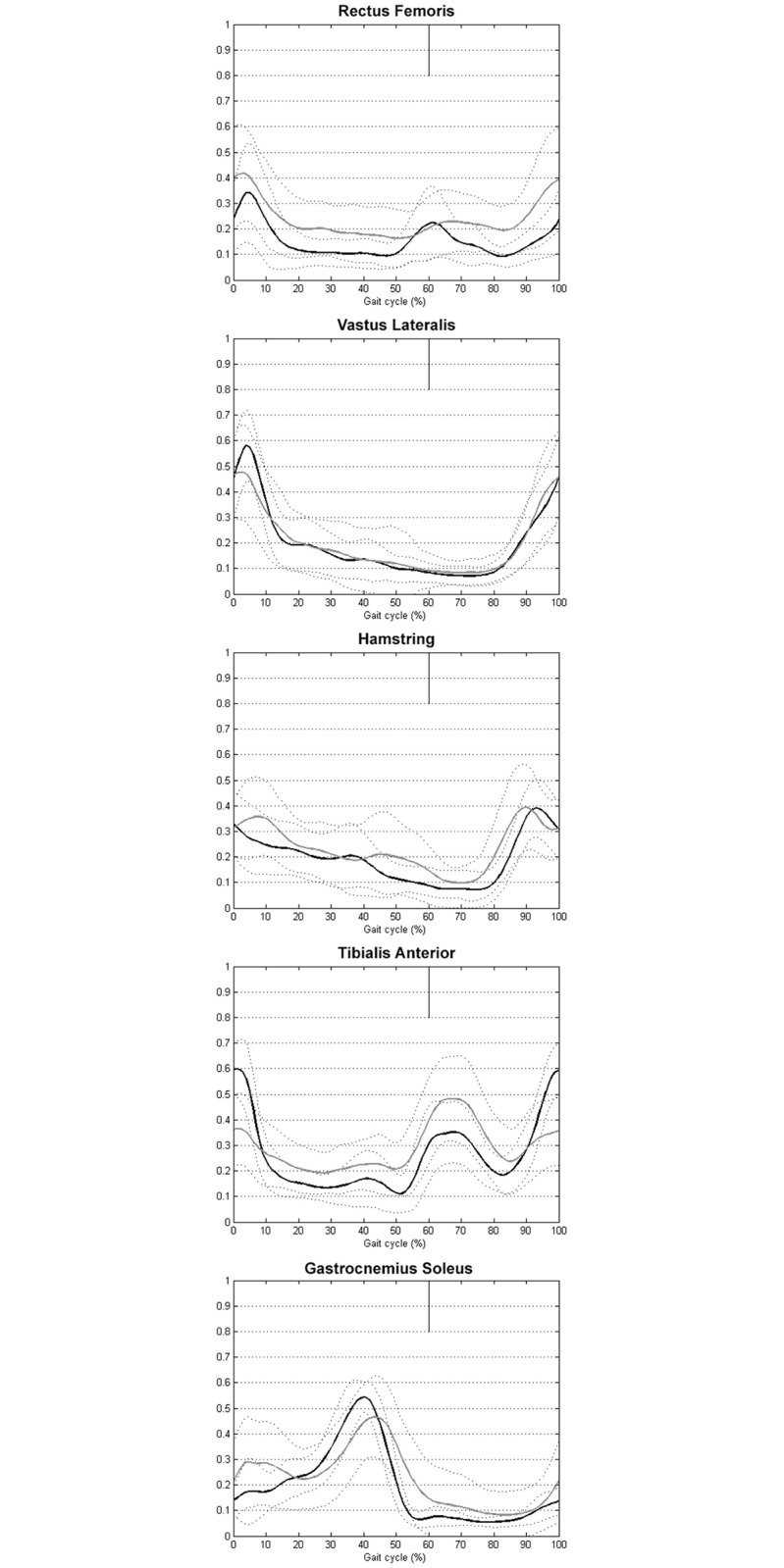
DMD group EMG profiles superimposed on control group EMG profiles. The percentage of the gait cycle is presented on the X-axis (0: heel strike, 100: the following ipsilateral heel strike) and the normalized amplitude of EMG activity on the Y-axis (0–1). The black (control group) and grey (DMD group) lines represent the mean linear envelope of muscle activation (mean of both sides). Dashed lines represent the mean ± standard deviation.

Qualitative analysis of the mean muscle activity (fig 2) showed that the percentage activation of the RF and HS was higher in the children with DMD for almost the whole of the gait cycle compared with the control children. The percentage activity of the TA was lower between 0–10% and 90–100% of the gait cycle and it was higher during the rest of the gait cycle, particularly during swing. The activity of the GAS was prolonged during stance (up to 60% of the gait cycle). Its activity was higher between 0 and 20% of the gait cycle (at the beginning of stance).

### Coactivation (table 4)

**Table 4:** Between group comparison of the co activation index (mean and standard deviation).

	DMD		Control
Muscle	Left	Right	Mean
RF / HS (N = 13 DMD)	0.15 (0.06) **	0.14 (0.06) **	0.09 (0.03)
VL / HS (N = 10 DMD)	0.13 (0.04) p = 0.56	0.12 (0.04) p = 0.91	0.12 (0.03)
TA / GAS (N = 13 DMD)	0.13 (0.04) **	0.14 (0.04) **	0.10 (0.03)

RF, rectus femoris; HA, hamstring; VL, vastus lateralis; TA, tibialis anterior; GAS, gastroctemius soleus.

^a^ p values were calculated using the Mann Whitney test (* p<0.05; ** p<0.01, *** p<0.001).

Proximally, there was significantly more coactivation between RF and HS during the whole of the gait cycle in the children with DMD (+0.06, p<0.05) but there was no significant difference between groups for the VL/HS pair. Distally, there was significantly more coactivation between TA and GAS in the children with DMD (+0.04, p<0.01), particularly during stance.

### Correlations between muscle activation, kinematics and functional status (table 5)

**Table 5:** Correlations between muscle activation, kinematics and functional status in children with DMD.

	Global indexes of muscle activity		Co activation index		
	Ker-EGI (N = 13)	Global EMG-PS (N = 16)	RF/HS (N = 16)	VL/HS (N = 13)	TA/GAS (N = 16)
Vignos functional scale	-0.53 p = 0.06	0.57 *	0.66 **	0.13 p = 0.66	0.15 p = 0.57
GDI	0.57 *	-0.59 *	-0.55 *	-0.49 p = 0.09	-0.62 *
GPS	-0.62 *	0.66 **	0.54 *	0.45 p = 0.13	0.61 *
Gait speed (m/sec)	0.001 p = 0.97	0.19 p = 0.48	-0.09 p = 0.74	-0.63 *	-0.12 p = 0.66

Ker-EGI, Ker-electromyography gait index; EMG-PS, electromyography profile score; GDI, gait deviation index; GPS, gait profile score; Values presented are the mean of both sides.

^a^ p values were calculated using the Spearman correlation test (* p<0.05; ** p<0.01, *** p<0.001).

The Vignos functional scale was significantly correlated with EMG-PS global in the children with DMD. Abnormal kinematic gait patterns (evaluated by the GDI and GPS) were also significantly correlated with global indexes of muscle activity (Ker-EGI and EMG-PS global). Proximally, the RF/HS coactivation index was significantly associated with a high Vignos score and pathological kinematic gait pattern (GDI, GPS). Distally, there were significantly correlations between the TA/GAS coactivation index and kinematic indexes. Walking speed was not statistically associated with muscle activity, except for the VL/HS coactivation index.

## Discussion

Altered muscle activity in children with DMD has already been reported in some studies [5,7,11] however, to our knowledge, no studies had previously quantified pathological muscle activity or coactivation, either globally or in individual muscles. This study used a recently developed process of EMG signal analysis to characterise patterns of muscle activity during gait in children with DMD with gait anomalies, and compared the patterns with those of typically developing children matched for age and gender. Activity (percentage activation amplitude) of the RF, HS and TA was increased and GAS activity was prolonged in stance compared with the control children. Activity of the TA was the most pathological, with increased activity throughout the gait cycle. Greater agonist/antagonist coactivation was found in the children with DMD, in both the proximal and distal muscles. These original results bring new knowledge of the pathophysiology of gait, particularly regarding adaptive muscle strategies used by children with DMD to compensate for muscle weakness during gait.

### Muscle hyper-activation

The results of this study confirmed quantitatively that the principal muscles of both lower limbs are hyper-active during gait in children with DMD [5,11]. The increased activity of TA, HS and RF is likely compensatory since the motor command is intact in these children. Children with DMD have 4 factors to compensate for: 1 –weakness of the muscles of the segment, as well weakness in the whole lower limb; 2 –imbalance of muscle strength within the segment [2]; 3- non-physiological joint positions such as equinus foot or excessive anterior pelvic tilt; and 4- loss of balance resulting from the 3 preceding factors. Furthermore, in the current study, we found that the amount of compensation (hyper-activity) was positively correlated to the severity of the functional status and gait pattern. In other words, muscle hyper-activity during gait increased with the progression of the disease. Particularly, the increase in percentage activation relative to the maximum recorded activation during gait could be the result of an increase in muscle fiber recruitment to compensate for weakness in the recorded muscle. Thus muscle weakness would be compensated for by an increase in muscle activity in children with DMD. This suggests that, to produce the same levels of force, children with DMD need greater muscle activity. However, the multifactorial nature of hyper-activity makes it difficult to establish a direct link between weakness and muscle hyper-activity.

### Function of the proximal muscles

The characteristic kinematic gait pattern of children with DMD has been suggested to be the result of the greater proximal than distal weakness, as well as the presence of muscle contractures in the more advanced stages of the disease [26]. The children with DMD in the present study had symmetrical weakness of the lower limbs that was predominant around the pelvis, as is classically described [2]. They did not have any muscle contractures (Table 2). It has been hypothetised that children with DMD bend their trunk backwards to keep the center of gravity behind the hips to prevent jack-knifing at the waist. It has also been suggested that to compensate for increasing knee extensor (quadriceps) weakness, they stabilize the knee by keeping the center of gravity in front of it [13]. The EMG activity of the RF and HS, as well as their coactivation, was significantly increased throughout almost all of the gait cycle, including swing. This demonstrates that the EMG activity of proximal muscles is abnormal in DMD, as has previously been suggested by Sutherland et Patte [5,11]. Since these muscles are the most affected in DMD, this confirms the hypothesis that hyper-activity compensates for weakness. However, the activity and coactivation of the VL was not abnormal. Weakness of the quadriceps group has been shown to be determining for gait in children with DMD [26]. Our results showed that abnormal activity in this group was particularly related to the RF, despite the fact that it is not the largest of the quadriceps muscles. The RF is the only bi-articular muscle of this group. It thus appears to have specific role as a stabiliser, by its capacity to keep the center of gravity behind the hips as well as in front of the knees. The different activities of these two quadriceps heads supports the idea that each has its own function. Their different functions could be studied by analysing the correlations between kinematics, dynamic EMG, and morphological (muscle MRI) and anatomopathological data.

### Function of the distal muscles

The activity of the TA in the children with DMD was the most abnormal of all the muscles studied. Paradoxically, this muscle was only moderately weak (mean 4.23/5) (Table 1), which does not support a relationship between weakness and hyper-activity. Moreover, there was a decrease in TA activity in the children with DMD at the end of the gait cycle compared with the control children. This hypo-activity could be anticipatory of foot-strike by the forefoot. This idea is reinforced by the fact that there was a relative hyper-activity of GAS during the initial part of the stance phase. This suggests that forefoot contact, which is a characteristic of gait in children with DMD [7], is an adaptive mechanism rather than a gait anomaly caused by pathological processes. This “voluntary” toe-gait could be a means to increase stability (body angle relative to the ground). These results invalidate current treatments which involve reducing equinus (surgery, splints etc.) in children with DMD.

### Coactivation

Agonist-antagonist coactivation was greater proximally (RF/HS) but also occurred distally (TA/GAS), with a significant correlation between the coactivation indexes of these muscles, and high Vignos score and pathological kinematic gait pattern (GDI, GPS). To our knowledge, this is the first study to quantify this phenomenon during gait in children with DMD. In healthy subjects, coactivations/co-contractions serve to stabilise joints, and there is a positive relationship between gait speed and co-contraction in all the segments of the lower limbs. The gait speed of the children with DMD in the present study was lower than normal, however they had higher levels of coactivation than the control children. Coactivation has been particularly studied in children with cerebral palsy [27,28] and has mostly been related to abnormal efferent commands, which is not the case in children with DMD. These patients develop an imbalance of muscle strength at every joint (hip flexors remain stronger than hip extensors; knee flexors (HS) remain stronger than knee extensors (quadriceps); ankle plantarflexors remain stronger than ankle dorsiflexors) [26]. Muscle weakness and imbalance at any joint leads to a loss of stability. Coactivation in children with DMD could be a strategy to increase stability around the knee and ankle. Coactivation of the knee muscles likely helps the child to maintain the center of gravity both behind the hips and in front of the knees. However, the predominance of this compensatory mechanism in the proximal muscles could have negative consequences, such as reducing the efficiency of joint motion and increasing the energy cost of gait [25].

### Limitations

The lack of statistical significance for some results (EMG-PS of the right RF and EMG-PS of the left GAS) is probably related to a lack of power due to the size of the sample of children with DMD. Thus, we were not able to assess the impact of critical factors that may influence DMD gait, such as corticotherapy (12 children out of 16) [29]. Of the 20 children with DMD whose gait was recorded, only 16 were included because the goal was to analyse a homogeneous group of children and gait patterns. A larger study with a follow-up of the children with DMD should improve the power of these preliminary results.

The limitations of EMG should be considered when interpreting the data, particularly interference from adjacent muscles and tissues, termed “cross-talk” [30]. This phenomenon prevented the recording of pelvic muscles which are particularly affected in DMD [26]. Moreover, in this disease, muscle fibers are replaced by fibrous and fatty tissue, altering the impedance of muscle tissue [31]. Variations in the structure and composition of the underlying soft tissue might alter the flow of the electrical current and, consequently, the voltage signals measured at the skin surface. The use of invasive intramuscular EMG electrodes was not considered appropriate for these children. The limitations of EMG should also be considered when interpreting the relationship between muscle weakness and activation: the EMG signal is a measure of muscle activation but does not measure the contraction/force produced.

The lower gait speed of the children with DMD (Table 2), principally the result of the muscle weakness, could have affected the results. However, Hof et al. and Schwartz showed a positive relationship between gait speed and amplitude of muscle activation with no significant effect on “timing” (percentage of the gait cycle) [32,33]. The muscle activity of the children with DMD in this study was similar or increased compared with the control children, despite their lower gait speed, thus reinforcing the theory of increased muscle activity in these children, despite the reduction in gait speed.

### Clinical impact

According to Petrof [34], dystrophin has a mechanical function on the membrane of muscle fibres, protecting the tissues from stress during muscle contraction. Hyper-activity of muscle fibres which lack dystrophin could thus affect the progression of the disease. These results support the controversial principle of avoiding muscle fatigue in children with DMD.

Based on the results of the present study, we propose that a reasonable rehabilitation objective would be to increase stability during gait, despite the muscle weakness. Increased stability would reduce muscle hyper-activity and thus prevent overworking of dystrophin-deficient muscles. Improving stability using a light dynamic or hinged orthosis, depending on the child’s gait pattern, might decrease the muscle hyperactivity. Future studies could evaluate the short-term effects of simple stabilising carbon ankle-foot orthoses or elastic bands on muscle activity and gait kinematics as well as on the maintenance of gait-capacity in the medium-term. Moreover, muscle hyperactivity could be used as an objective outcome measure of the impact of pharmacological treatments on DMD muscle (corticotherapy as well as other potential drug therapies).

Also with the aim to increase stability during gait, physiotherapy could work to increase balance and to concentrically strengthen muscles such as TA. However, in a systematic review in 2013 [35], Gianola et al underlined the lack of evidence of effectiveness on stability and the potentially deleterious effects on strength, highlighting the need for more studies regarding this issue.

## Conclusion

The results of this study showed hyper-activity of the RF and HS, and particularly of the TA associated with coactivation in both the proximal and distal segments of the lower limbs. Hyper-activity was not found in the VL and GAS, showing specific adaptations of the RF, HS and TA during gait. Hyper-activity and coactivation probably compensate for instability caused by the muscle weakness, however they may have negative consequences on the muscles and increase energy cost of gait.

Simple therapeutic solutions such as targeted physiotherapy or ankle-foot orthoses could improve stability and reduce abnormal muscle activity. However, more evidence is necessary for the formal recommendation of these interventions.

## Abbreviations

BMI: Body Mass Index
CHRU: Centre Hospitalier Régional Universitaire
CI: Coactivation Index
DMD: Duchenne muscular Dystrophy
EMG: Electromyography
EMGs: Surface Electromyography
EMG-PS: Electromyography-profil score
EMG-PS StP: Electromyography-profil score stance phase
EMG-PS Swp: Electromyography-profil score swing phase
GDI: Gait Deviation Index
GM: Gluteus Maximus
GPS: Gait Profile Score
HS: Hamstring
Ker-EGI: Ker-electromyography gait index
3D: three-dimensionnal
RF: Rectus Femoris
RMSE: Root Mean Square Error
GAS: Gastrocnemius Soleus
TA: Tibialis Anterior
VL: Vastus Lateralis
